# Supplementary material for: Correcting glucose-6-phosphate dehydrogenase deficiency with a small-molecule activator
Source: Nat Commun. 2018 Oct 2;9:4045. doi: 10.1038/s41467-018-06447-z (PMC6168459; doi:10.1038/s41467-018-06447-z)
Supplement: Supplementary file 1 — Supplementary Information [file 41467_2018_6447_MOESM1_ESM.pdf]

Supplementary Information

Correcting glucose-6-phosphate dehydrogenase deficiency with a small-molecule activator

Hwang et al.

Supplementary Figures

Figure S1

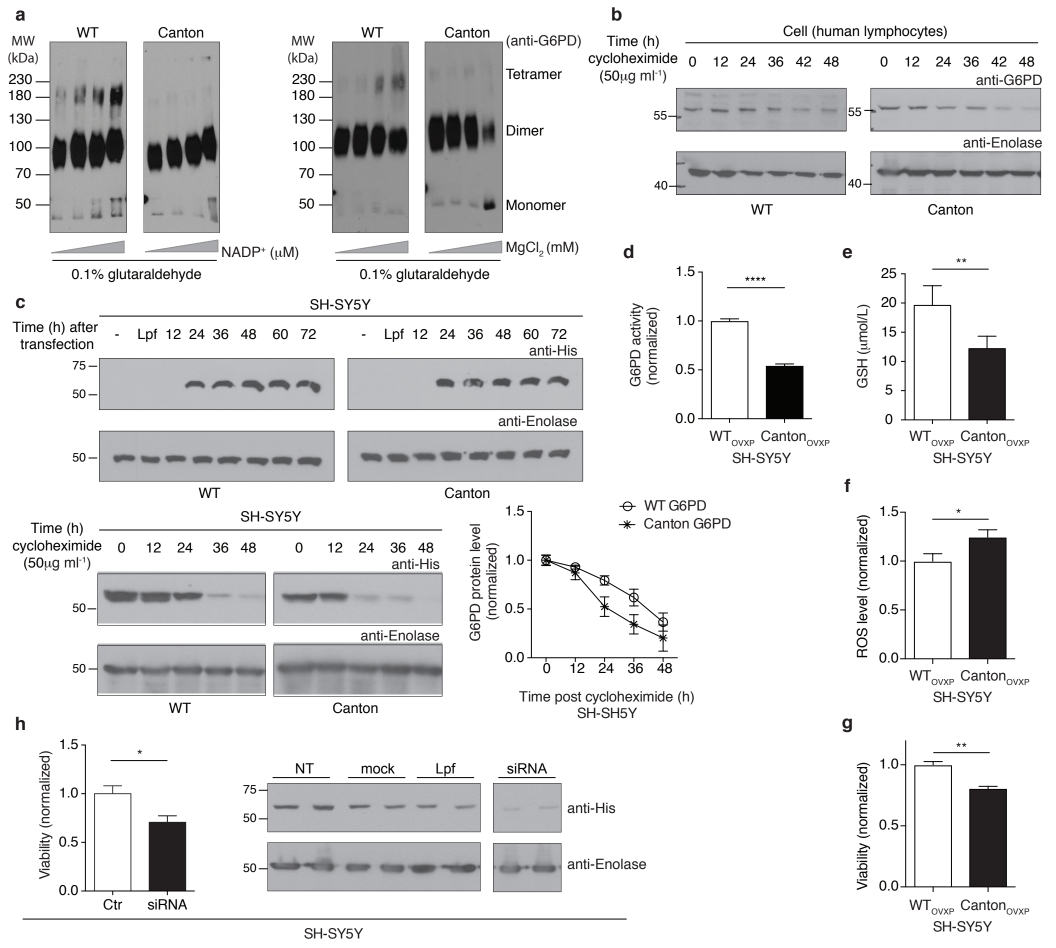

Supplementary Figure 1. Canton variant (R459L) is biochemically different from WT G6PD. **a** Canton G6PD forms less tetramer as compared to WT G6PD in the presence of $NADP^+$ or $MgCl_2$, when cross-linked by 0.1% glutaraldehyde. Some dissociation of oligomers was observed at a high concentration of cofactors possibly due to NADPH production as a result of enzyme reaction with either bacterial $NADP^+$ or G6P or both copurified with the enzymes. **b** Representative Western blot showing turnover rate of G6PD protein in lymphocytes after inhibition of *de novo* protein biosynthesis with cycloheximide. **c** (Top) In SH-SY5Y neuronal cells, the transient expression of WT and Canton G6PD lasted up to 72 hours after transfection. (Bottom) Overexpressed Canton variant was degraded faster than WT G6PD following cycloheximide (50 μg mL^{-1}) treatment. **d, e, f, g** The lysates with the Canton variant showed lower G6PD enzymatic activity (n=3, $^{****}p < 0.0001$). The cells expressing the Canton variant showed lower GSH levels (n=4, $^{**}p=0.0098$), higher ROS levels (n=6, $^{*}p=0.04$), and lower cell viability (n=4, $^{**}p=0.0014$), when they were cultured under stress induced by serum starvation. **h** Knockdown of G6PD by siRNA decreased cell viability in SH-SY5Y cells (n=4, $^{*}p=0.033$). G6PD level was confirmed by Western blot. Error bars represent mean ± SEM. Statistical differences were calculated by two-tailed unpaired Student's *t*-test. Molecular weight marker is provided on the left of the blots. NT: no treatment; MW: molecular weight; WT: wild-type; Ctr: control; Lpf: lipofectamine; ovxp: overexpression.

Figure S2

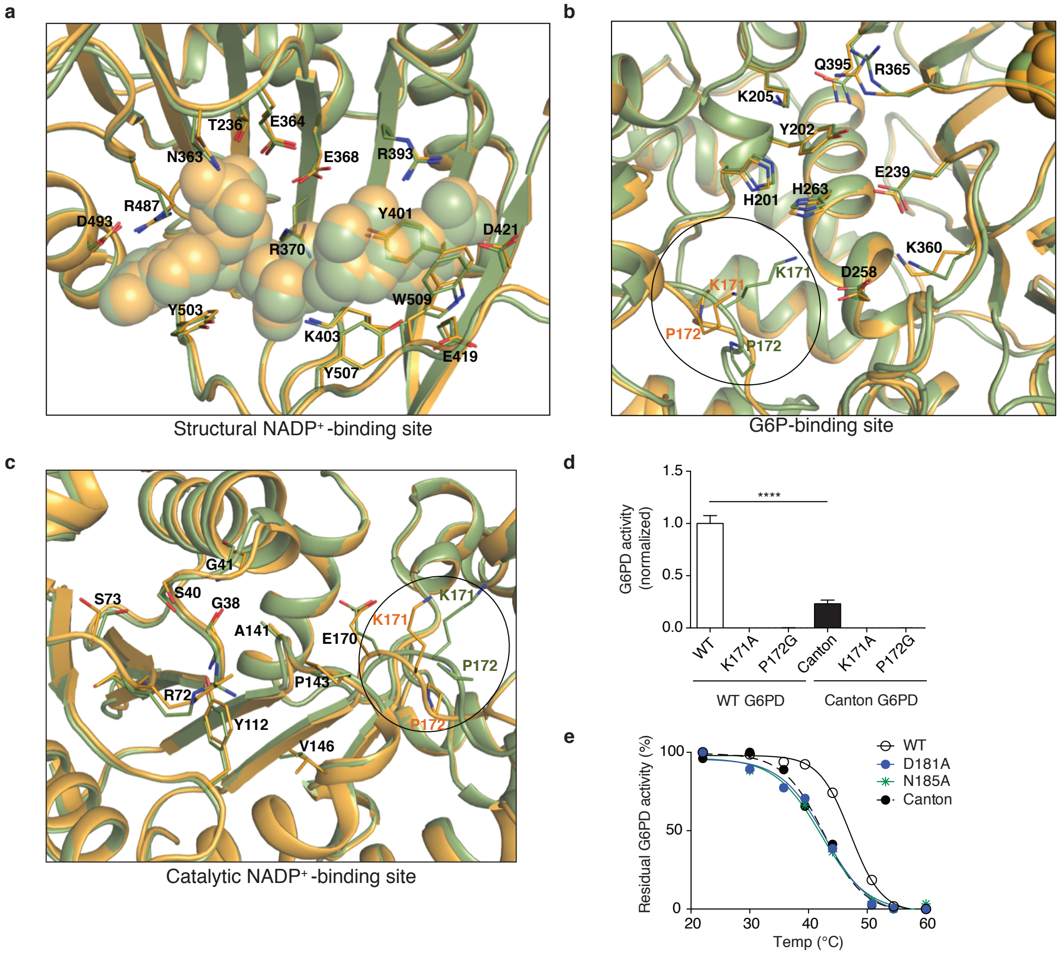

Supplementary Figure 2. Some residual disorder is found in G6P- and catalytic NADP$^+$-binding sites of Canton G6PD as compared with WT G6PD. **a** Structural NADP$^+$-binding site is well-conserved in the structures of WT G6PD and Canton G6PD, shown in green and orange, respectively. Side chains of residues involved in binding to NADP$^+$ are illustrated by sticks. **b, c** Orientation of side chains of essential residues around G6P- and catalytic NADP$^+$-binding sites. Key residues, K171 and P172, involved in positioning of G6P and NADP$^+$ in their binding pockets, were circled. **d** Catalytic activity of K171A and P172G mutant enzymes (n=4, $^{****}p<0.0001$, two-tailed unpaired Student's *t*-test). **e** Thermal inactivation curves of WT G6PD, Canton G6PD and mutant enzymes of R459-interacting residues, D181 and N185. $T_{1/2}$ values were summarized in Fig. 1D and Fig. 2C. WT: wild-type.

Figure S3

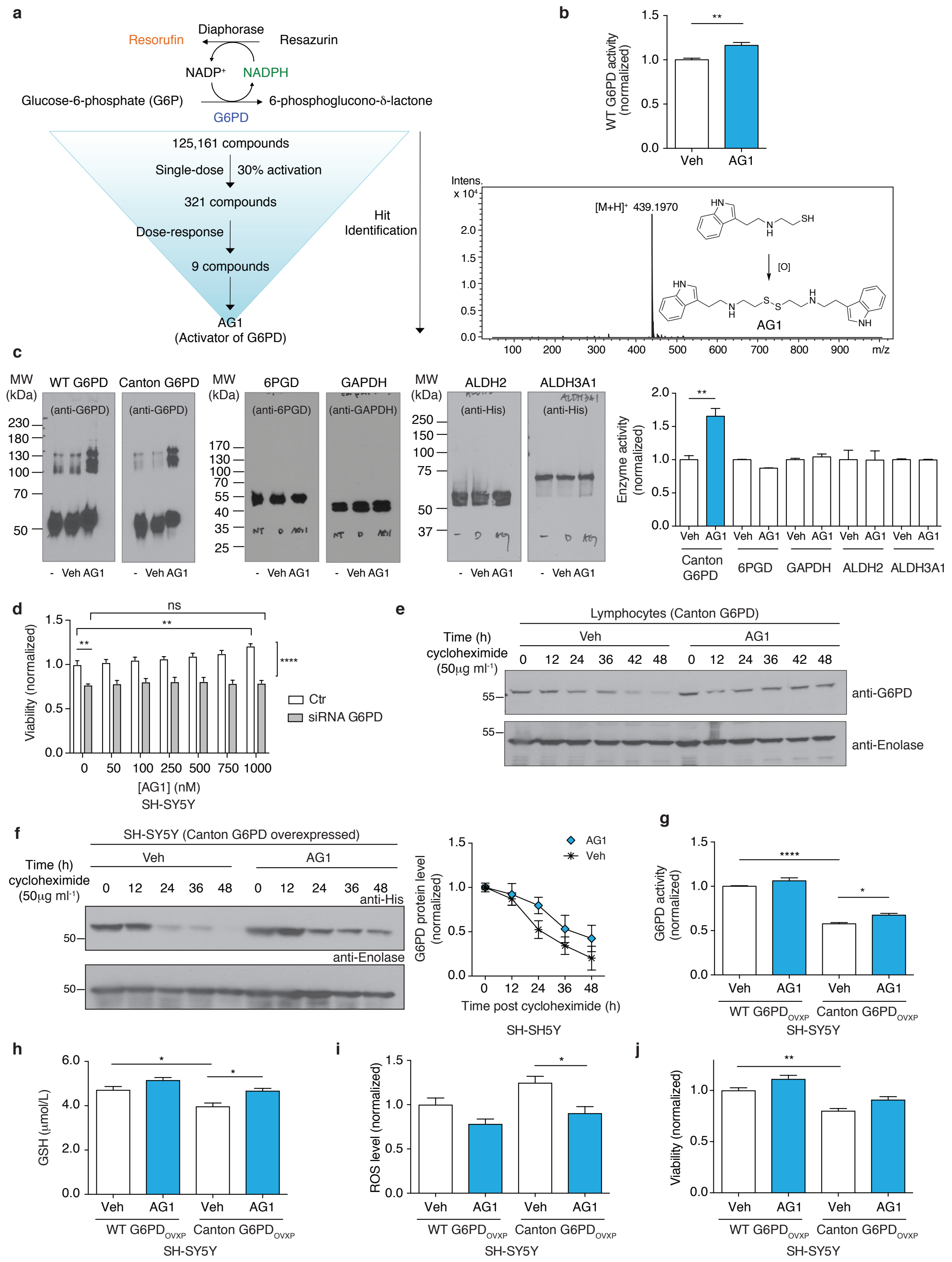
a
Diaphorase
Resorufin
Resazurin
NADP+
NADPH
Glucose-6-phosphate (G6P)
6-phosphoglucono-δ-lactone
G6PD
125,161 compounds
Single-dose
30% activation
321 compounds
Dose-response
9 compounds
AG1
(Activator of G6PD)
Hit Identification
b
WT G6PD activity (normalized)
Veh
AG1
[M+H]+ 439.1970
AG1
c
WT G6PD
Canton G6PD
6PGD
GAPDH
ALDH2
ALDH3A1
Enzyme activity (normalized)
d
Viability (normalized)
Ctr
siRNA G6PD
[AG1] (nM)
SH-SY5Y
e
Lymphocytes (Canton G6PD)
anti-G6PD
anti-Enolase
f
SH-SY5Y (Canton G6PD overexpressed)
anti-His
anti-Enolase
G6PD protein level (normalized)
Time post cycloheximide (h)
SH-SH5Y
g
G6PD activity (normalized)
h
GSH (μmol/L)
i
ROS level (normalized)
j
Viability (normalized)
WT G6PDOVXP
Canton G6PDOVXP
SH-SY5Y

Supplementary Figure 3. AG1 (activator of G6PD) induces biochemical changes of the Canton variant. **a** Schematic high-throughput screening assay for G6PD activators. AG1 among 9 selected compounds reproducibly generated consistent results between assays. The structure of the active form of AG1 [connected via a disulfide bridge] and its mass spectrum [calculated for $C_{24}H_{30}N_4S_2$ 438.1912, found 439.1970] are provided on the right of the scheme. [The nuclear magnetic resonance spectroscopic analysis is provided in Supplementary Note 1.] **b** Activation of WT G6PD by AG1 (n=5, **p=0.0022, two-tailed unpaired Student's *t*-test). **c** AG1 selectively promoted dimerization of G6PD among other NAD- or $NADP^+$-dependent dehydrogenases (*n=2*) and enhanced only G6PD activity (n=3, **p=0.001, two-tailed unpaired Student's *t*-test). Each enzyme activity was normalized to the Veh-treated enzyme activity. **d** Viability of SH-SY5Y cells was enhanced by AG1 treatment, when cells were under stress by serum starvation. siRNA-directed knockdown of G6PD reduced cell viability, which was unaffected by AG1 treatment (n=4, **p<0.01, ****p<0.0001*,* ns: not statistically significant, two-way ANOVA). **e**, **f** Representative Western blots showing improved protein stability by AG1 in lymphocytes carrying the Canton variant and in SH-SY5Y neuroblastoma cells overexpressing the Canton variant, when *de novo* protein biosynthesis was inhibited by cycloheximide (50 μg ml^{-1}) (n=3). **g**, **h, i** AG1 slightly increased G6PD enzymatic activity in lysates of SH-SY5Y cells overexpressing WT G6PD and Canton variant (n=3, ****p<0.0001, *p=0.0419, one-way ANOVA) and GSH levels in culture (n=3, *p<0.05*,* one-way ANOVA) and mildly reduced ROS levels (n=6, *p=0.0122, one-way ANOVA), when the cell stress was induced by serum starvation. **j** AG1 mildly (albeit not statistically significant) improved cell viability under the same condition (n=4, **p=0.0021, one-way ANOVA). Measurements were normalized to the parameters in WT $G6PD_{ovxp}$ Veh-treated condition. 100 μM of AG1 was used for *in vitro* assays, and 1 μM of AG1 was used for cell-based assays. 5% DMSO (stock concentration) was used as vehicle (Veh). Cells were subjected to serum starvation (50-75%) for 48 hours. Error bars represent mean ± SEM. Molecular weight marker is provided on the left of the blots. Ctr: control; ovxp: overexpression; MW: molecular weight; WT: wild-type; Veh: vehicle; Intens.: intensity.

Figure S4

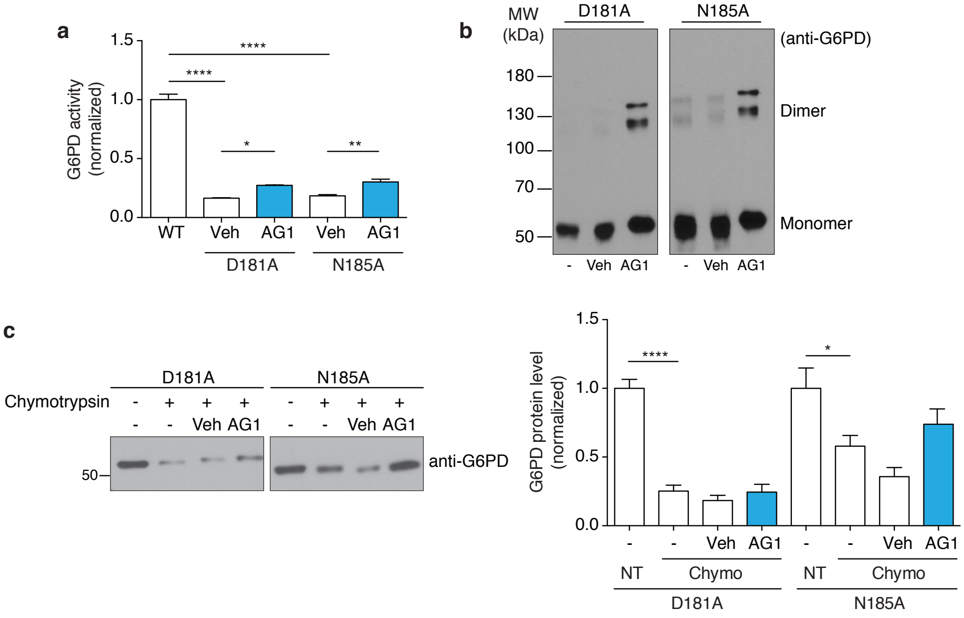

Supplementary Figure 4. AG1 affects the mutations of R459-interacting residues, D181A and N185A. **a**, **b** AG1 increased activity and promoted dimerization of these mutant enzymes (activity assay: n=3 for WT G6PD; n=4 for D181A and N185A, ****p<0.0001, *p=0.0108, **p=0.0063, one-way ANOVA; Western blot assay: n=3). The activity was normalized to WT G6PD activity. **c** AG1 mildly prevented mutant enzymes from proteolytic degradation by chymotrypsin (n=4 for D181A; n=3 for N185A, ****p<0.0001, *p=0.0255, one-way ANOVA). The protein level was normalized to the level of the enzyme under non-treated (NT) condition. 100 μM of AG1 was used for *in vitro* assays. 5% DMSO (stock concentration) was used as vehicle (Veh). Error bars represent mean ± SEM. Molecular weight marker is provided on the left of the blots. Chymo: chymotrypsin; MW: molecular weight; WT: wild-type; Veh: vehicle.

Figure S5

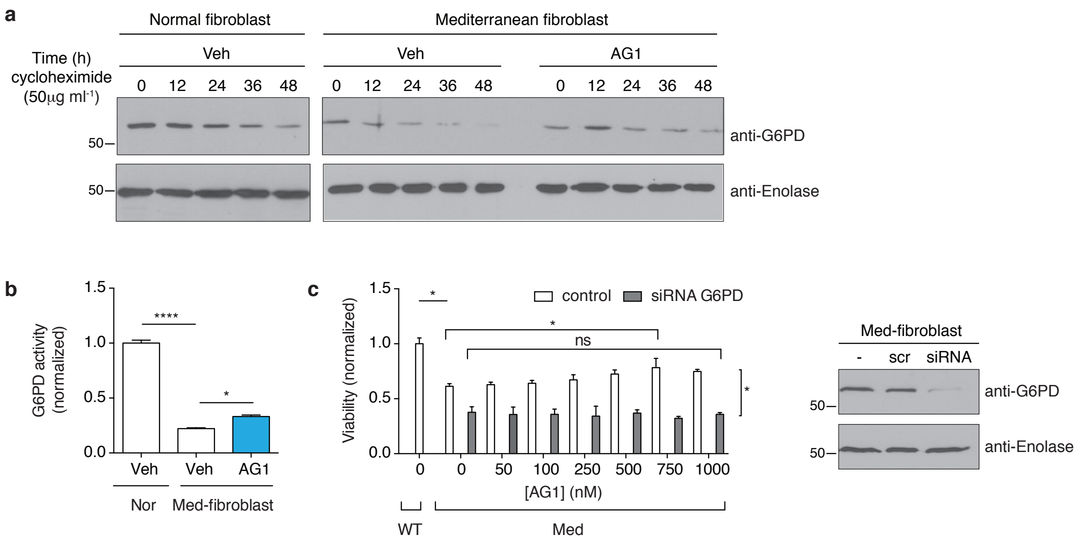

Supplementary Figure 5. Fibroblasts carrying Mediterranean mutation (S188F) are also affected by AG1 treatment. **a** Representative Western blots showing improved protein stability by AG1 (1 μM) in fibroblast cells derived from a subject carrier of Mediterranean variant, when *de novo* protein biosynthesis was inhibited by cycloheximide (50 μg ml^{-1}) (*n=4*). **b** AG1 increased G6PD activity in cell lysates (n=3, $^{****}p<0.0001$, $^{*}p=0.012$, one-way ANOVA). **c** AG1 increased cell viability of fibroblast cells carrying the Mediterranean variant, whereas there was no change in viability when Mediterranean G6PD was knocked down by siRNA (n=2, $^{*}p<0.05$, two-way ANOVA). Western blot image showed G6PD protein levels after siRNA-mediated G6PD knockdown. 100 μM of AG1 was used for *in vitro* assays, and 1 μM of AG1 was used for cell-based assays. 5% DMSO (stock concentration) was used as vehicle (Veh). Cells were subjected to serum starvation for 48 hours. Error bars represent mean ± SEM. Molecular weight marker is provided on the left of the blots. WT: wild-type; Nor: normal fibroblast; Med: Mediterranean fibroblast; scr: scrambled siRNA; Veh: vehicle; ns: not statistically significant.

Figure S6

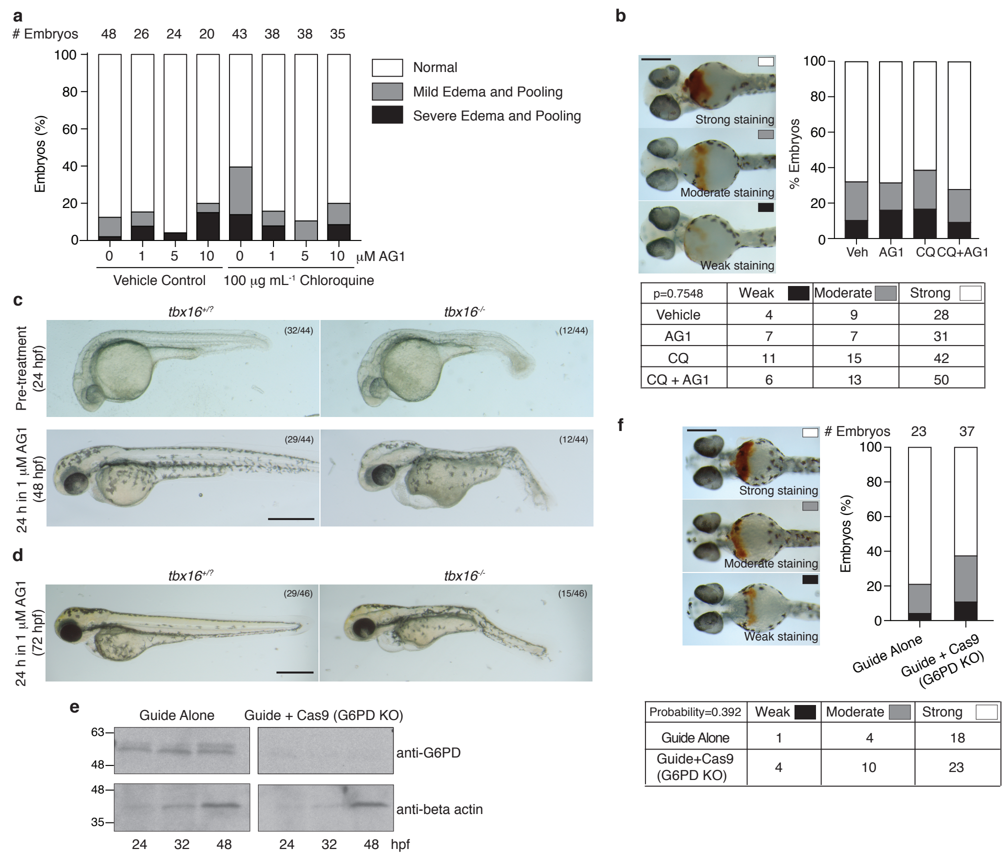

p=0.7548	Weak	Moderate	Strong
Vehicle	4	9	28
AG1	7	7	31
CQ	11	15	42
CQ + AG1	6	13	50

Probability=0.392	Weak	Moderate	Strong
Guide Alone	1	4	18
Guide+Cas9 (G6PD KO)	4	10	23

Supplementary Figure 6. AG1 attenuates ROS-induced pericardial edema in a G6PD-dependent manner. **a** AG1 is not toxic to zebrafish embryos at concentrations <10 µM. Pericardial edema induced by chloroquine treatment was partially attenuated by AG1 treatment. Embryos were treated at 24 hpf with indicated concentrations of AG1 for 24 hours. Severity of pericardial edema is indicated by color schemes provided on the right of the graph. **b** Ventral views of whole-mount *o*-dianisidine staining of WT embryos at 48 hpf. Representative staining is shown on the left for embryo scoring; distribution of staining shown on the right. Raw counts used for chi-square analysis and calculated p value are included in table below. Scale bar: 300 µm. **c, d** Genetic loss of *tbx16* function led to mesodermal defects in embryos obtained by incrossing *tbx16* mutant heterozygotes. Pre-treatment at 24 hpf with 1 µM AG1 for 24 hours did not prevent pericardial edema that developed at 48 hpf in *tbx16* mutants (**c**) nor was edema rescued at 72 hpf (**d**), when treated with AG1 at 48 hpf. Phenotypic ratio is indicated at the upper right corner of each image. Embryo orientation is lateral view, anterior left. Scale bar: 500 µm. **e** Embryos injected with sgRNA (Guide Alone) against exon 10 of *g6pd* and sgRNA+Cas9 protein (Guide+Cas9, G6PD KO (knockout)) were pooled at indicated times and analyzed for the G6PD protein level by Western blot. **f** Ventral views of whole-mount *o*-dianisidine staining of embryos injected with sgRNA (Guide Alone) and sgRNA+Cas9 protein (Guide+Cas9, G6PD KO) at 48 hpf. Scale bar: 300 µm. Representative staining is shown on the left for embryo scoring; distribution of staining shown on the right. Raw counts used for Fisher exact test and calculated exact probability are included in table below. Molecular weight marker is provided on the left of the blots. CQ: chloroquine; WT: wild-type; KO: knockout; hpf: hours post fertilization; Veh: vehicle.

Figure S7

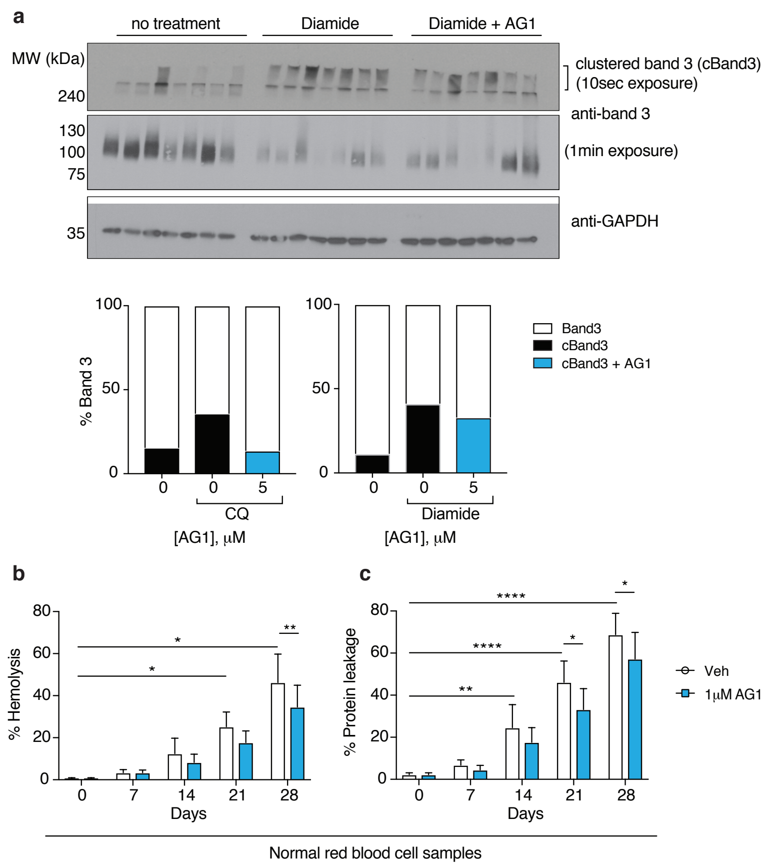

Supplementary Figure 7. AG1 reduces hemolysis of human erythrocytes. **a** (Top)Western blot data showing band 3 clustering under stress condition induced by diamide (1 mM) treatment. Each lane represents one individual sample. (Bottom) Corresponding quantification data are provided below (please see Fig. 5e for Western blot data showing band 3 clustering with chloroquine treatment). **b, c** Time-course of hemoglobin release and protein leakage from 5% erythrocyte suspension while they were stored at refrigerated temperature for 28 days (see Fig. 5f, g. n=13 independent blood samples, $^{*}p<0.05$, $^{**}p<0.01$, $^{****}p<0.0001$, two-way ANOVA). Molecular weight marker is provided on the left of the blots. CQ: chloroquine; Veh: vehicle; cBand3: clustered band 3; MW: molecular weight.

Figure S8 (source data)

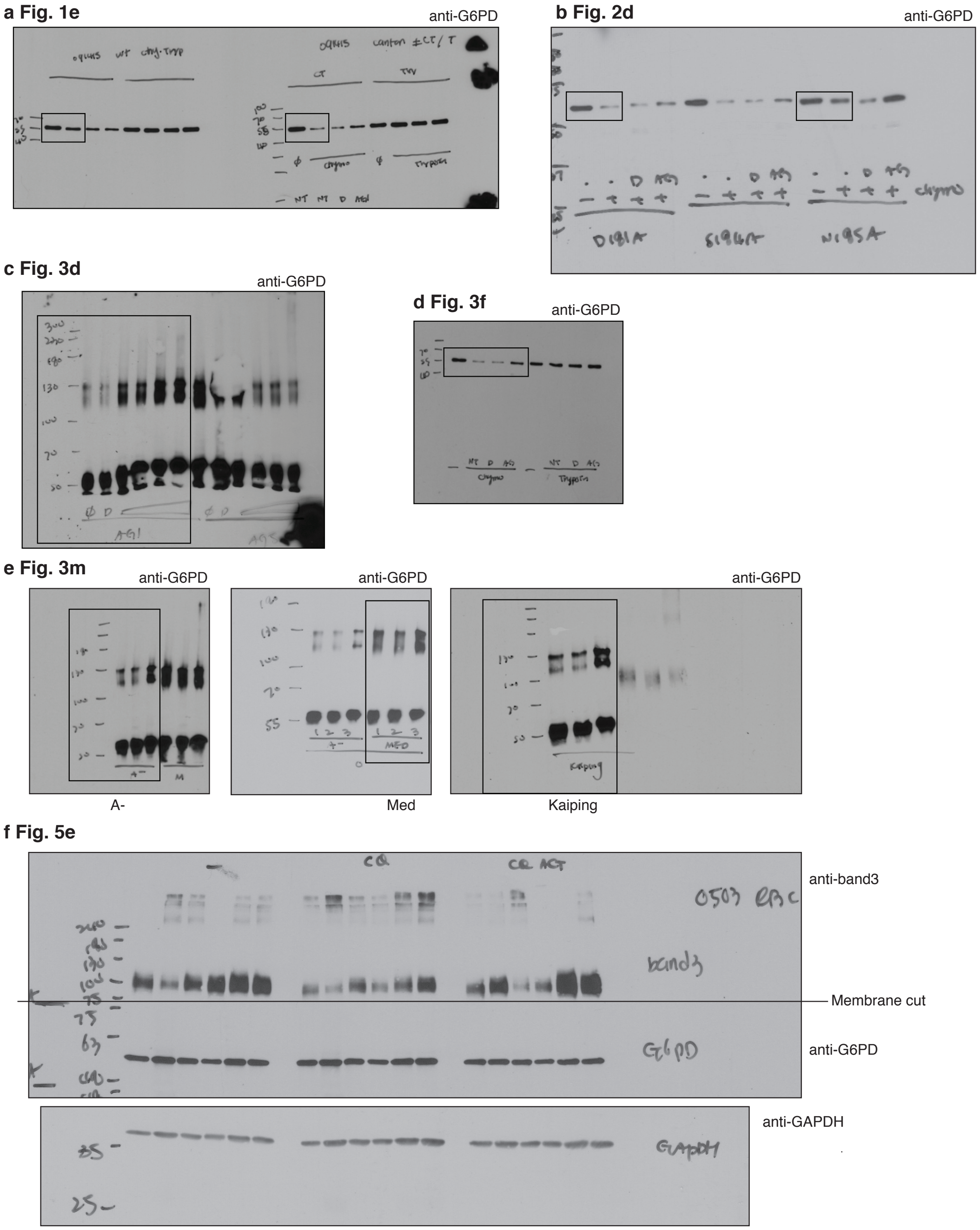

Supplementary Figure 8. Original source data of the blot images used in Fig. 1e, Fig. 2d, Fig. 3d, Fig. 3f, Fig. 3m and Fig. 5e.

Figure S9 (source data)

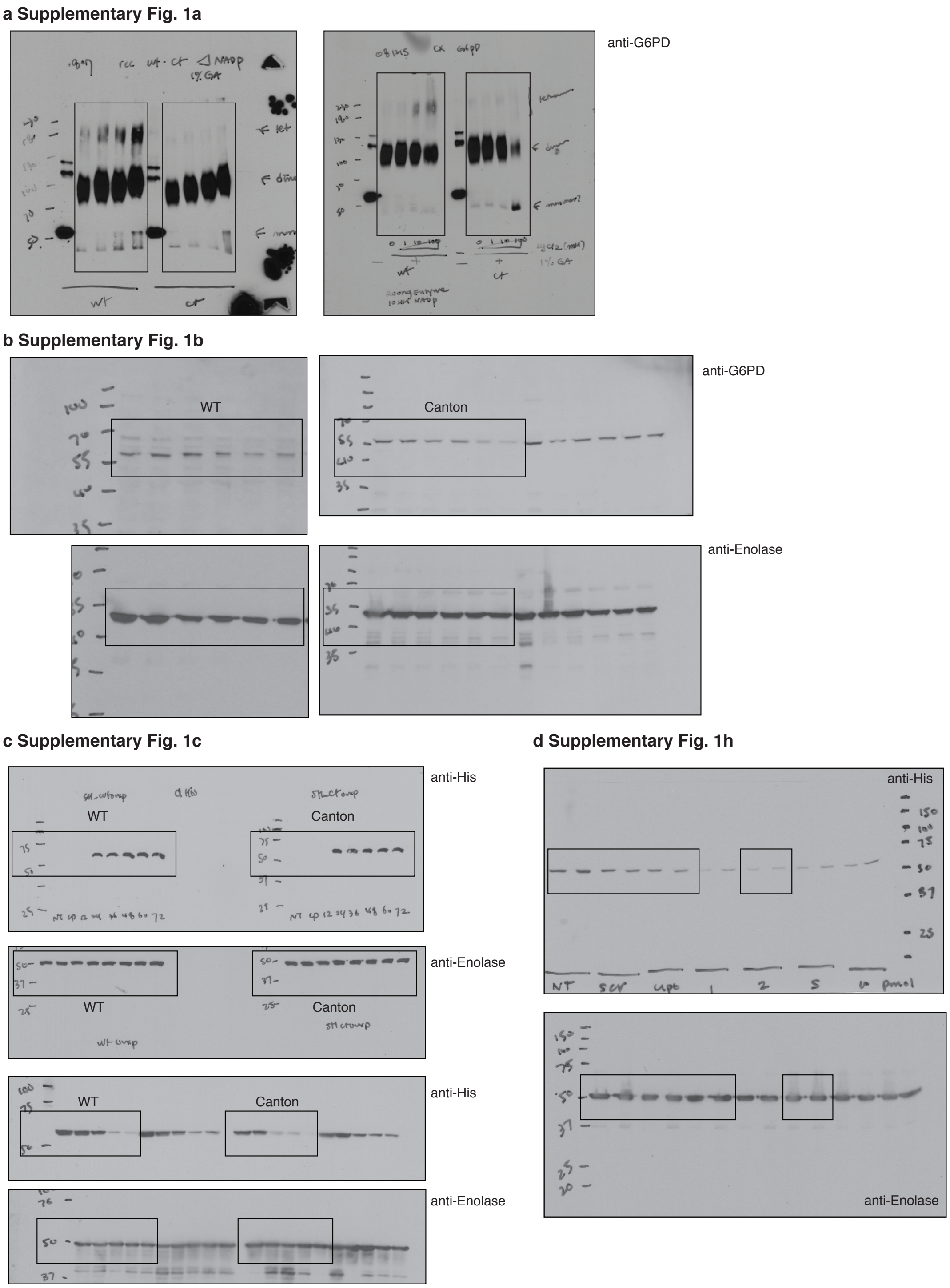

Supplementary Figure 9. Original source data of the blot images used in Supplementary Fig. 1a, Supplementary Fig. 1b, Supplementary Fig. 1c and Supplementary Fig. 1h.

Figure S10 (source data)

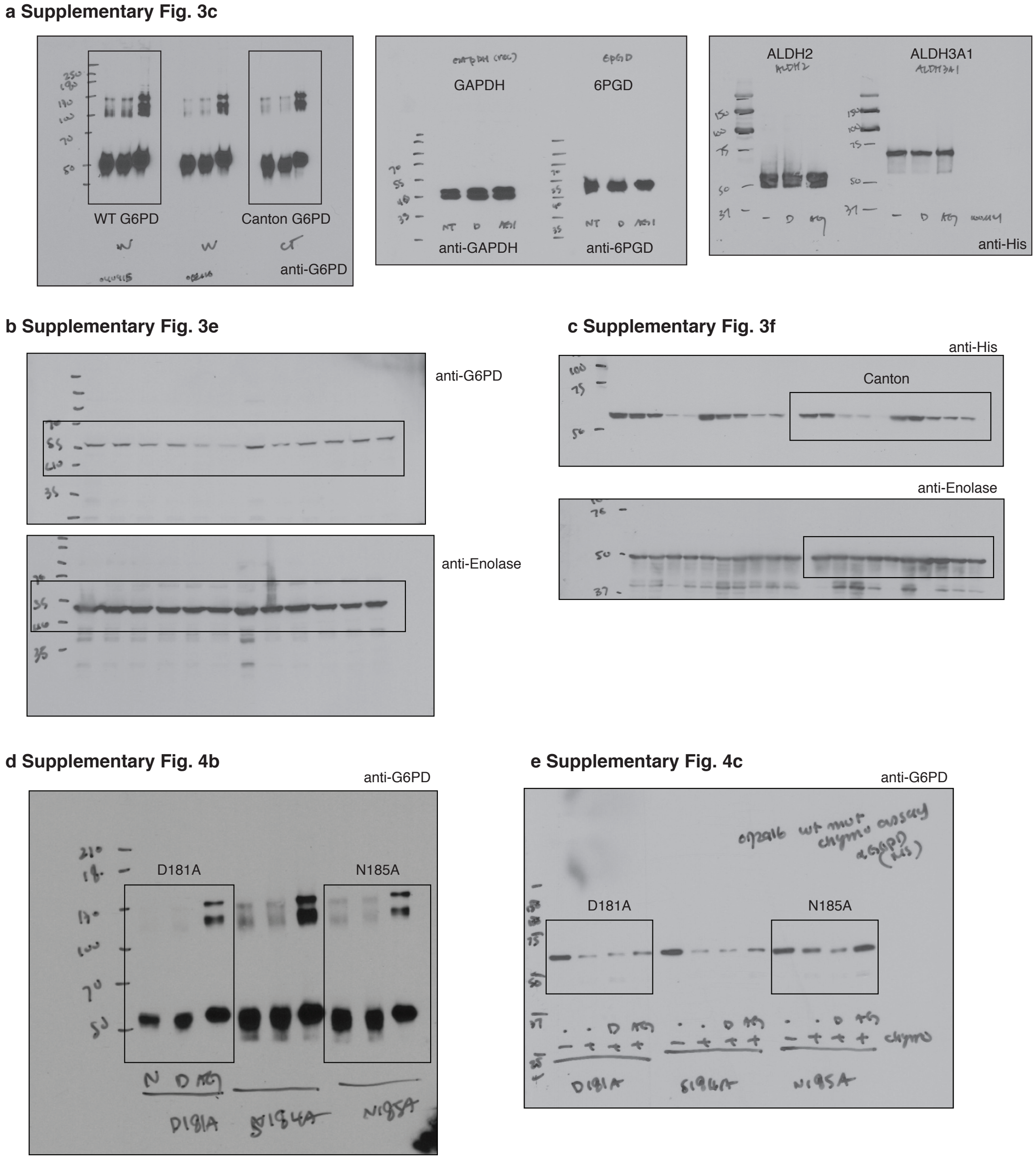

Supplementary Figure 10. Original source data of the blot images used in Supplementary Fig. 3c, Supplementary Fig. 3e, Supplementary Fig. 3f, Supplementary Fig. 4b and Supplementary Fig. 4c.

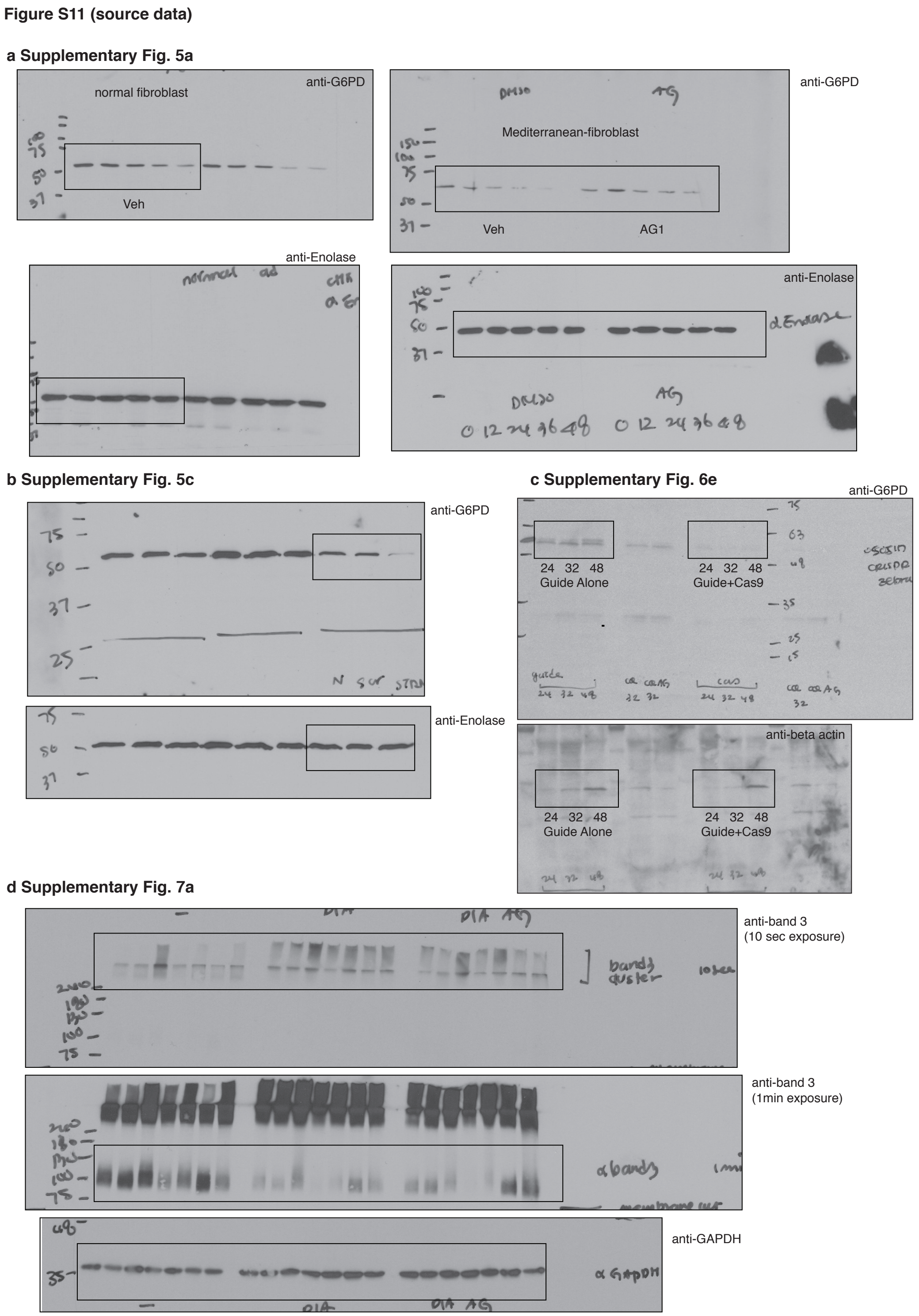

Supplementary Figure 11. Original source data of the blot images used in Supplementary Fig. 5a, Supplementary Fig. 5c, Supplementary Fig. 6e and Supplementary Fig. 7a.

Supplementary Table 1.

Primer sets for mutagenesis and cloning for *in vitro* assays and cell-based assays.

Canton (R459L)	CGCCAGGCCTCAAGGAGCTCGTCGC<br>GCGACGAGCTCCTTGAGGCCTGGCG
A- (V68M, N126D)	AACGGGCATAGCCCATGATGAAGGTGTTTTCGG<br>CCGAAAACACCTTCATCATGGGCTATGCCCGTT<br>CCAGGTGGAGGGCATCCATGTGGCTGTTGAG<br>CTCAACAGCCACATGGATGCCCTCCACCTGG
Mediterranean (S188F)	CTCACGGAACAGGGAGAAGATGTGGTTGGACAG<br>CTGTCCAACCACATCTTCTCCCTGTTCCGTGAG
Kaiping (R463H)	GTGGGGTGAAAATATGCCAGGCCTCACGG<br>CCGTGAGGCCTGGCATATTTTCACCCCAC
D181A	GTTGGACAGCCGGGCAGAGCTCTGCAG<br>CTGCAGAGCTCTGCCCGGCTGTCCAAC
N185A	GGGAGGAGATGTGGGCGGACAGCCGGTCAG<br>CTGACCGGCTGTCCGCCCACATCTCCTCCC
K171A	GTCCCTCCCGAAGGGGGCCTCCACGATGATGCG<br>CGCATCATCGTGGAGGCCCCCTTCGGGAGGGAC
P172G	GGTCCCTCCCGAAGCCCTTCTCCACGATGA<br>TCATCGTGGAGAAGGGCTTCGGGAGGGACC

Supplementary Note 1

Characterization of AG1:

^{1}H NMR (500 MHz, DMSO-d_6) δ 10.80 (s, 2H), 7.51 (d, J = 7.9 Hz, 2H), 7.33 (d, J = 8.1 Hz, 2H), 7.14 (s, 2H), 7.05 (t, J = 7.6 Hz, 2H), 6.96 (t, J = 7.5 Hz, 2H), 3.13 – 2.56 (m, 18H).

^{13}C NMR (126 MHz, DMSO) δ 136.24, 127.23, 122.58, 120.82, 118.28, 118.12, 112.38, 111.32, 49.63, 48.01, 38.36, 25.47.

HRMS (m/z): calculated for $C_{24}H_{30}N_4S_2$, 438.1912; found, 439.1970 ($M+H^+$).
